# Precautions for avoiding pulmonary morbidity after esophagectomy

**DOI:** 10.1002/ags3.12354

**Published:** 2020-06-08

**Authors:** Naoya Yoshida, Kazuto Harada, Masaaki Iwatsuki, Yoshifumi Baba, Hideo Baba

**Affiliations:** ^1^ Department of Gastroenterological Surgery Graduate School of Medical Sciences Kumamoto University Kumamoto Japan

**Keywords:** esophageal cancer, precaution, pulmonary complication, surgery

## Abstract

Pulmonary morbidity is the most common complication after esophagectomy. Importantly, it is the main cause of surgery‐related mortality and possibly adversely affects the long‐term outcome after surgery in patients with esophageal cancer. There is considerable accumulated evidence on multidisciplinary approaches to reduce post‐operative pulmonary morbidity. A comprehensive review of the precautionary measures that have so far been shown to be effective in previous literature is of utmost importance. We herein update and summarize the perioperative and surgical approaches to diminish pulmonary morbidity. Pre‐operative smoking cessation, respiratory rehabilitation, maintaining oral hygiene, perioperative nutritional intervention, enforcement of less invasive surgery, perioperative administration of steroid, and total management by a multidisciplinary team could be the key factors contributing to reduction in pulmonary morbidity.

## Graphical abstract

Pulmonary morbidity is the most common complication and the main cause of surgery‐related mortality after esophagectomy. A comprehensive review of the precautionary measures that have so far been shown to be effective in previous literature is of utmost importance. We update and summarize the perioperative and surgical approaches to diminish pulmonary morbidity based on recently published evidence.

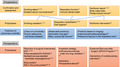

## 1 INTRODUCTION

Esophagectomy is the mainstay of multidisciplinary therapies for resectable esophageal cancer. However, esophagectomy is associated with higher morbidity and mortality rates compared to other gastrointestinal surgeries.^1^, ^2^, ^3^ Pulmonary morbidity is one of the most common morbidities after esophagectomy. Although the occurrence of pulmonary morbidity is gradually reducing, recent studies reported that it still occurs in 16%‐23% of cases.^2^, ^3^, ^4^, ^5^ Pulmonary morbidity is also a major cause of hospital mortality^6^ and might be an independent risk factor for poorer long‐term survival.^7^, ^8^ Thus, identifying the risk factors and preventing pulmonary morbidity is important to improve surgical outcomes in patients with resectable esophageal cancer. A comprehensive review of the precautionary measures that have so far been shown to be effective in previous literature is of utmost importance.

**Figure 1:**
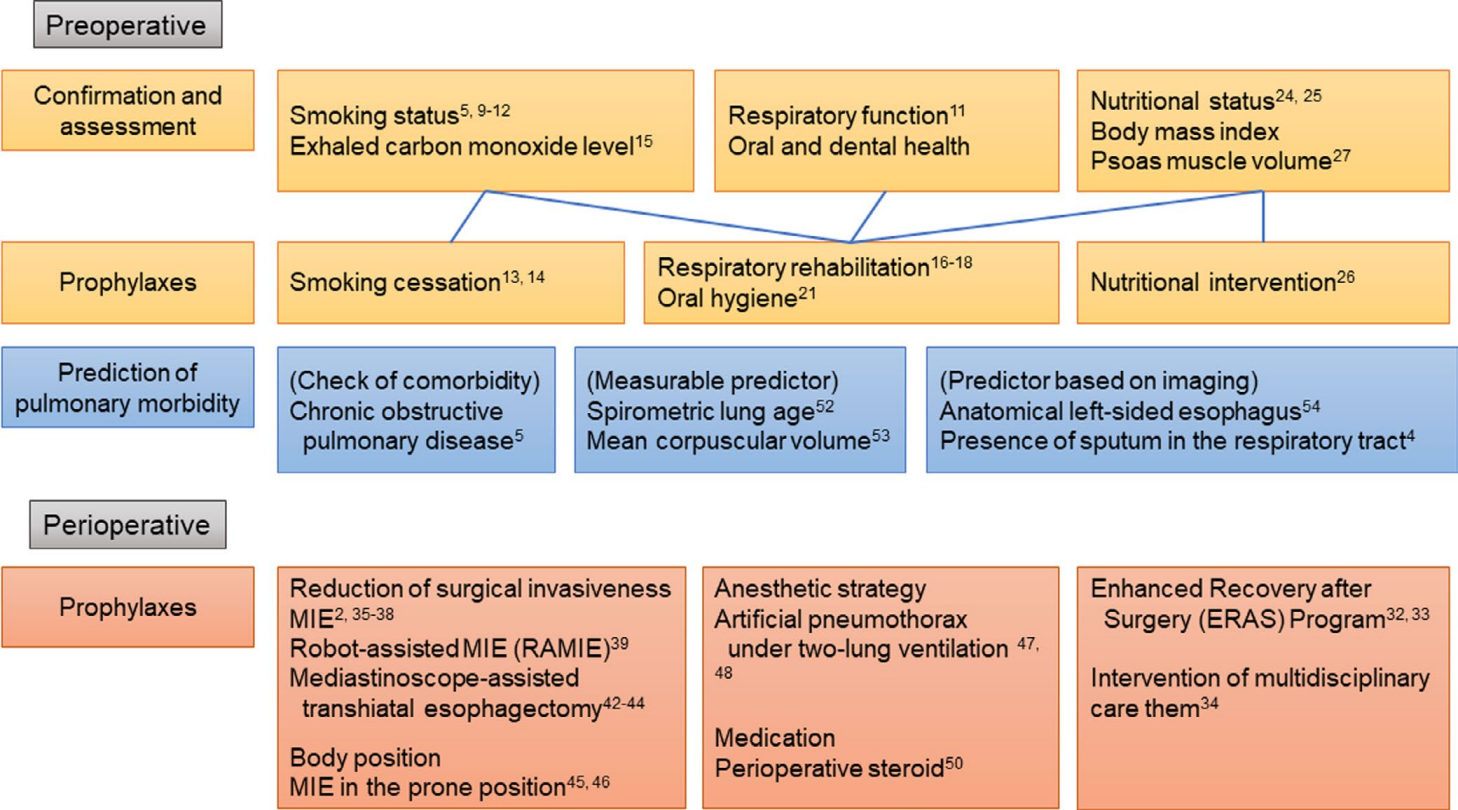
Summary of precautions for avoiding pulmonary morbidity after esophagectomy. MIE, minimally invasive esophagectomy

Herein, we update and summarize methods that contribute to reducing pulmonary morbidity after esophagectomy based on recently published evidence. Representative data are summarized in Figure 1.

## 2 SMOKING CESSATION

Smoking habit is one of the significant risk factors for pulmonary morbidities.^5^, ^9^, ^10^, ^11^, ^12^ Notably, current smoking at the time of surgery contributes considerable risk.^5^, ^11^ Previous studies suggested that chances of occurrence of pneumonia increased as the duration of pre‐operative smoking cessation became shorter.^13^, ^14^ The minimum duration for which patients should stop smoking before surgery to reduce pulmonary morbidity has not been established. We have reported that the duration of pre‐operative smoking cessation should be more than at least 30 days to reduce post‐esophagectomy pulmonary morbidity.^13^ It is sometimes difficult to determine whether or not the patient has truly stopped smoking before surgery. In such cases, checking the exhaled carbon monoxide level might be useful in estimating current smoking status and the risk of post‐esophagectomy morbidities.^15^

## 3 RESPIRATORY REHABILITATION

Impaired respiratory function correlated with frequent pulmonary morbidities after esophagectomy.^11^ Existence of comorbid chronic obstructive pulmonary disease could also be a risk factor.^5^ Several studies indicated the usefulness of pre‐operative respiratory rehabilitation to improve respiratory function.^16^, ^17^ A prospective randomized controlled trail (RCT) suggested that intensive pre‐operative respiratory rehabilitation significantly diminished post‐operative pulmonary morbidities.^18^

## 4 ORAL HYGIENE

Bacteria from the oral biofilms are aspirated into the respiratory tract, which could cause their colonization and progression of pneumonia.^19^, ^20^ Maintaining oral hygiene is important to prevent pulmonary complication. Notably, patients who have antibiotic‐resistant microbes in the saliva might have a significant risk of post‐operative pulmonary morbidity.^21^ A prospective study suggested that pre‐operative dental brushing (5 times a day) could significantly reduce post‐operative pneumonia and tracheostomy after esophagectomy.^22^ A multi‐center retrospective study indicated that pre‐operative oral care by dentists and dental hygienists could significantly reduce the incidence of post‐esophagectomy pneumonia.^23^

## 5 PRE‐OPERATIVE NUTRITIONAL INTERVENTION

Pre‐operative malnutrition confers a risk of pulmonary complications after esophagectomy.^24^, ^25^ A meta‐analysis suggested that immunoenhancing nutritional intervention could reduce post‐operative pulmonary complications.^26^ Shichinohe et al described the importance of preoperative psoas muscle volume and body mass index to select patients requiring preoperative nutritional intervention and physical rehabilitation.^27^ To maintain or improve pre‐operative nutritional status, several novel approaches are being investigated. Ghrelin administration is one of the potential candidates that might help minimize the deterioration of nutritional status^28^ and reduce the incidence of pneumonia.^29^ Although esophageal stenting during pre‐operative therapy for patients with stenosis is also effective in maintaining nutritional status,^30^ it might cause a decline in the R0 resection rate.^31^

## 6 ENHANCED RECOVERY AFTER SURGERY (ERAS) PROGRAM

The ERAS program originally aimed to facilitate patient recovery following major surgery, mainly colorectal surgery. The ERAS program contains various items, such as antimicrobial prophylaxis, nasogastric intubation, perioperative fluid management, post‐operative analgesia, post‐operative nutritional care, and early mobilization. Several meta‐analyses suggested that the ERAS program could significantly reduce the incidence rate of pulmonary morbidity after esophagectomy.^32^, ^33^

## 7 MANAGEMENT BY MULTIDISCIPLINARY PERIOPERATIVE CARE TEAM

Peri‐operative management via a multidisciplinary peri‐operative care team, which consists of a nurse, a pharmacist, a physical therapist, a dentist, a dietician, a liaison psychiatry team, a dysphagia rehabilitation team, and a surgeon, might contribute to reducing post‐operative pulmonary morbidities.^34^ A multidisciplinary care team could reduce the surgeon's workload. Moreover, systematic and cooperative interaction among professionals could minimize the risk of post‐operative complications.

## 8 SURGICAL STRATEGY

Minimally invasive esophagectomy (MIE) and hybrid MIE are considered less invasive compared to open esophagectomy. Several RCTs and studies with large real‐world cohorts suggested that using these approaches could contribute to reducing pulmonary morbidity.^2^, ^35^, ^36^, ^37^, ^38^ A robot‐assisted MIE (RAMIE) could also reduce pulmonary morbidities when compared to that seen with open esophagectomy.^39^ Incidence rates of pulmonary morbidities following RAMIE and MIE are reportedly equivalent.^40^, ^41^ Mediastinoscope‐assisted transhiatal esophagectomy without transthoracic manipulation could further contribute to reducing pulmonary morbidities than that with MIE and RAMIE. Incidence rate of pulmonary morbidities in mediastinoscope‐assisted transhiatal esophagectomy was reported to range from 0% to 9.5%.^42^, ^43^, ^44^ This strategy requires an accomplished surgeon who has experience in performing this complicated surgery. However, this procedure is expected to be an alternative to the currently performed esophagectomy, which has a high incidence rate of pulmonary morbidities.

The body position during esophagectomy could affect post‐operative morbidity after MIE.^45^ A propensity score‐matched analysis suggested that thoracoscopic esophagectomy in the prone position correlates with a lower incidence rate of pulmonary morbidity than that with the left‐lateral position.^46^

## 9 ANESTHETIC AND ANALGESIC STRATEGY

Anesthetic and analgesic strategy might affect the incidence rate of post‐esophagectomy pulmonary morbidity. Thoracoscopic esophagectomy using artificial pneumothorax under two‐lung ventilation could potentially reduce intraoperative lung damage and also maintain hemodynamics and oxygenation during surgery.^47^ This method generated no excessive increase in airway pressure and provided a good surgical view without excluding the lung, which might contribute to reducing pulmonary morbidity.^47^, ^48^ Post‐operative analgesia is important in preventing pulmonary morbidities, because it helps patients to easily cough out sputum. A meta‐analysis suggested that analgesic strategy (comparison of systemic vs epidural analgesia) did not affect the incidence rate of pulmonary morbidity.^49^

## 10 PERIOPERATIVE ADMINISTRATION OF STEROID AND NEUTROPHIL ELASTASE INHIBITOR

The use of steroids might contribute to reducing pulmonary complications. A meta‐analysis suggested that perioperative glucocorticoid administration reduced pulmonary morbidity along with organ failure and cardiovascular morbidity.^50^ A meta‐analysis indicated the usefulness of perioperative administration of neutrophil elastase inhibitor in preventing post‐esophagectomy acute lung injury.^51^ However, the study contained several biased retrospective studies with small numbers of patients. Thus, further investigations are required to establish the effectiveness of this agent with regard to post‐esophagectomy pulmonary morbidities.

## 11 MARKERS TO PREOPERATIVELY PREDICT THE OCCURRENCE OF PULMONARY MORBIDITY

Several measurable markers for estimating the occurrence of pulmonary morbidity have been reported. Spirometric lung age, calculated as [0.036 × body height (cm) − 1.178 − FEV1 (L)]/0.028 for men and [0.022 × body height (cm) − 0.005 − FEV1 (L)]/0.022 for women, might reflect impaired respiratory function and is correlated with the incidence of post‐esophagectomy pneumonia.^52^ High mean corpuscular volume, which is etiologically associated with high tobacco consumption, might predict post‐esophagectomy pulmonary morbidities.^53^

Novel predictors based on imaging have also been reported. Anatomical left‐sided esophagus in the thorax on computed tomography (CT) is associated with surgical difficulties in MIE and contributes to increased pulmonary morbidity.^54^ Asymptomatic sputum in the respiratory tract on preoperative CT could predict the occurrence of post‐esophagectomy pneumonia.^4^ When these findings are identified preoperatively, appropriate prophylactic approaches could be considered during esophagectomy.

## 12 CONCLUSION

Recent advances in surgical strategy and perioperative management contribute to reducing pulmonary morbidity after esophagectomy. Accumulation of the latest evidence on risk assessment and multidisciplinary approaches for the prevention of pulmonary morbidity is integral to further improve surgical outcomes after esophagectomy.

## DISCLOSURE

Drs. Naoya Yoshida belongs to the department supported by Chugai Pharmaceutical Co., Ltd and Yakuruto Honsya Co., Ltd, but has no conflicts of interest regarding this research. Dr Yoshifumi Baba belongs to the department supported by Ono Pharmaceutical Co., Ltd, but has no conflicts of interest regarding this research. Hideo Baba and the other co‐authors (KH and MI) have no conflicts of interests or financial ties to disclose.
